# Transcriptome and Metabolome Integration Provides New Insights Into the Regulatory Networks of Tibetan Pig Alveolar Type II Epithelial Cells in Response to Hypoxia

**DOI:** 10.3389/fgene.2022.812411

**Published:** 2022-01-21

**Authors:** Yanan Yang, Haonan Yuan, Xuanbo Liu, Zhengwen Wang, Yongqing Li, Yue Ren, Caixia Gao, Ting Jiao, Yuan Cai, Shengguo Zhao

**Affiliations:** ^1^ College of Animal Science and Technology, Gansu Agricultural University, Lanzhou, China; ^2^ Xinjiang Academy of Animal Sciences, Ürümqi, China; ^3^ Academy of Agriculture and Animal Husbandry Sciences, Institute of Animal Husbandry and Veterinary Medicine, Lhasa, China; ^4^ State Key Laboratory of Veterinary Biotechnology, Harbin Veterinary Research Institute, Chinese Academy of Agricultural Sciences, Harbin, China; ^5^ College of Grassland Science, Gansu Agricultural University, Lanzhou, China

**Keywords:** hypoxia, ATII cells, swine, MAPK pathway, mRNA-metabolite network, glycolysis

## Abstract

Tibetan pigs show a widespread distribution in plateau environments and exhibit striking physiological and phenotypic differences from others pigs for adaptation to hypoxic conditions. However, the regulation of mRNAs and metabolites as well as their functions in the alveolar type II epithelial (ATII) cells of Tibetan pigs remain undefined. Herein, we carried out integrated metabolomic and transcriptomic profiling of ATII cells between Tibetan pigs and Landrace pigs across environments with different oxygen levels to delineate their signature pathways. We observed that the differentially accumulated metabolites (DAMs) and differentially expressed genes (DEGs) profiles displayed marked synergy of hypoxia-related signature pathways in either Tibetan pigs or Landrace pigs. A total of 1,470 DEGs shared between normoxic (TN, ATII cells of Tibetan pigs were cultured under 21% O_2_; LN, ATII cells of Landrace pigs were cultured under 21% O_2_) and hypoxic (TL, ATII cells of Tibetan pigs were cultured under 2% O_2_; LL, ATII cells of Landrace pigs were cultured under 2% O_2_) groups and 240 DAMs were identified. Functional enrichment assessment indicated that the hypoxia-related genes and metabolites were primarily involved in glycolysis and aldosterone synthesis and secretion. We subsequently constructed an interaction network of mRNAs and metabolites related to hypoxia, such as guanosine-3′, 5′-cyclic monophosphate, Gly-Tyr, and phenylacetylglycine. These results indicated that mitogen-activated protein kinase (MAPK) signaling, aldosterone synthesis and secretion, and differences in the regulation of *MCM* and adenosine may play vital roles in the better adaptation of Tibetan pigs to hypoxic environments relative to Landrace pigs. This work provides a new perspective and enhances our understanding of mRNAs and metabolites that are activated in response to hypoxia in the ATII cells of Tibetan pigs.

## Introduction

Tibetan pigs are well adapted to high-altitude environments and primarily live in semi-agricultural and semi-pastoral areas with an average elevation of 2,500–4,300 m on the Qinghai-Tibet Plateau in Southwest China, as an ideal model for investigating the biological mechanisms of hypoxic adaptation (Groenen et al., 2012; Shang et al., 2020). In particular, hypoxia poses a major barrier to life, as pulmonary respiratory function is typically injury by a modest decline in oxygen (Nathan et al., 2019; Grimmer et al., 2021). Gas exchange in the lung occurs within alveoli, which are mainly composed of alveolar type I and II epithelial (ATI and ATII) cells. The lung is a unique physiological environment from a metabolic perspective, as it constitutes the barrier between external air and the pulmonary vasculature in the body (Liu and Summer 2019). Metabolomics is expected to provide a reliable assessment of the physiological status of cells and can reveal change in cell metabolic pathways under hypoxia via the systematic study of small-molecule metabolite profiles (Gunda et al., 2018). At present, lung epithelium is derived from pluripotent stem cells, which have been extensively characterized to discover targets for new therapeutics for hypoxic disease (Herriges and Morrisey, 2014). The hypoxic phenotype is characterized by a shift away from primary reliance on oxidative phosphorylation to enhanced glycolysis for the maintenance of homeostasis (Lottes et al., 2014). ATII cells have a highly oxidative metabolic phenotype; they can consume lactate for oxidative ATP production and synthesize the majority of lipid components as a site of lactate consumption to produce pulmonary surfactants (Lottes et al., 2014). Mammalian targets of the cAMP signaling pathway, hypoxia inducible factor (HIF) pathway, AMP-activated kinase pathway, and many other signaling pathways are involved in mediating cellular metabolic homeostasis and the response to hypoxia (Zhang et al., 2018b; Li et al., 2019; Jones et al., 2020). For example, ATII cells are a heterogeneous cell population that contributes to alveolar maintenance and through metabolic and molecular adaptation in the alveolus, to maintain bioenergetic homeostasis after experiencing insults in a hypoxic environment. These cell play roles in processes including fluid transport and homeostasis (Matalon, 1991), pulmonary surfactant production (Guillot et al., 2013), and may serve as progenitors that undergo significant regeneration and transdifferentiation to repopulate ATI cells (Cardoso and Whitsett, 2008; Hoffman and Ingenito, 2012). Recent studies of lung development under hypoxia have generated novel insights into the function and molecular regulatory pathways of different cell lineages in the lung. Although ATII cells may repair lung damage under hypoxia (Lottes et al., 2014), the effects of O_2_ limitation on the mRNA and metabolic pathways necessary to maintain cellular energy in ATII cells have not been studied extensively. This project aimed to reveal the network and intercellular signal transduction pathways that contribute to energy homeostasis by identifying DEGs and specific metabolic processes via 2D analyses of the transcriptome and metabolome using primary ATII cells of Tibetan pigs cultured in normoxic and hypoxic conditions.

## Materials and Methods

### Ethics Statement

All animal experiments were conducted according to the guidelines for the care and use of experimental animals established by the Ministry of Science and Technology of the People’s Republic of China (Approval number: 2006–398). The procedures for animal care were approved by the Gansu Agricultural University Animal Care and Use Committee of Gansu Agricultural University, and all experiments were conducted in accordance with approved relevant guidelines and regulations.

### *In vitro* Hypoxia Model in ATII Cells

Lung tissues were collected from healthy newborn male piglets (Tibetan pigs and Landrace pigs) at 7 days of age. The lung tissues were perfused with normal saline to flush out any remaining blood and soaked in PBS buffer containing penicillin and streptomycin. ATII primary cells were isolated as described previously (Wang et al., 2016), with minor modifications. ATII cells were cultured in complete medium (DMEM) at 37°C under normoxic conditions, with 21% O_2_, 5% CO_2_, and 79% N_2_. Hypoxic conditions were achieved by culturing cells in a hypoxic atmosphere containing 2% O_2_, 5% CO_2_, and 98% N_2_ under hypoxic conditions (Bactal 2 gaz; Air Liquide, France).

### RNA Extraction and Transcriptome Sequencing

ATII cells were collected at 48 h under 21% O_2_ (TN, LN) or 2% O_2_ (TL, LL) conditions, and three of nine replicates were selected from each group. RNA was extracted using TRIzol reagent kit (Invitrogen, Carlsbad, CA, United States). Second-strand cDNA synthesis was performed with DNA polymerase I, RNase H, dNTPs (dUTP instead of dTTP) and buffer. PCR amplification, sequencing library generation and subsequent sequencing were carried out using the Illumina HiSeqTM 4,000 platform (or other platforms) by Gene Denovo Biotechnology Co. (Guangzhou, China).

### Transcriptome Analysis

Raw data were filtered with fastp (version 0.18.0, https://github.com/OpenGene/fastp) (Chen et al., 2018b) to trim adapter sequences and obtain high-quality clean reads. The short-reads alignment tool Bowtie2 (version 2.2.8, http://bowtie-bio.sourceforge.net/bowtie2/index.shtml) (Langmead and Salzberg, 2012) was used to map reads to the ribosomal RNA (rRNA) database, and the remaining reads were further used for the assembly and analysis of the transcriptome. An index of the reference genome was built by using HISAT (http://ccb.jhu.edu/software/hisat/index.shtml) (Kim et al., 2015) software v2.0.4, and clean reads were mapped to the *Sus scrofa* genome in RefSeq (*Sus scrofa* 11.1). The fragments per kilobase of transcript per million mapped reads (FPKM) method was used to calculate gene expression levels with StringTie (https://ccb.jhu.edu/software/stringtie/index.shtml) (Pertea et al., 2015) software, and differentially expressed genes (DEGs) were defined according to a |log2 (fold change)| ≥ 1 and a false discovery rate (FDR) < 0.05. DEGs were analyzed according to Gene Ontology (GO) categories with an online tool (http://www.geneontology.org/), and the Kyoto Encyclopedia of Genes and Genomes (KEGG) pathways (http://www.genome.jp/kegg/pathway.html) were represented in scatter plots.

Eight mRNAs were randomly selected and quantified using qRT-PCR to validate the accuracy of the sequencing data. The primers used for qPCR were designed and synthesized by Qingke Biological Company (Xi’an, China) (Supplementary Table S1). To normalize sample differences, the β-actin gene served as an internal control, and the 2^−ΔΔCT^ method was used to calculate relative expression levels. *p*<0.05 was considered significant.

### Metabolite Extraction

Six of nine replicates were set for each group using the same protocol. Metabolite extraction, endogenous metabolite identification and data processing were carried out by Genedenovo Tech Co., Ltd. (Guangzhou, China) using liquid chromatography–mass spectroscopy (LC-MS). The samples were freeze dried in the same proportions, after which 1,000 µl of methanol (−20°C) was added to the lyophilized powder, and the samples were centrifuged at 12,000 rpm for 10 min at 4°C. A 450 μl aliquot of the supernatant was then concentrated by drying under vacuum, dissolved in 150 μl of a 2-chlorobenzalanine (4 ppm) 80% methanol solution, and filtered through a 0.22 µm membrane. A 20 µl aliquot of each sample was used for quality control (QC) analysis, and the remaining samples were used for LC-MS analysis.

### Metabolome Analysis by Liquid Chromatography–Mass Spectroscopy (LC–MS)

UHPLC-MS/MS analyses were performed using a Vanquish UHPLC system (ThermoFisher, Germany) coupled with an Orbitrap Q ExactiveTM HF-X mass spectrometer (Thermo Fisher, Germany) in Gene Denovo Co., Ltd. (Guangzhou, China). Samples were injected onto a Hypesil Gold column (100 × 2.1 mm, 1.9 μm) using a 17 min linear gradient at a flow rate of 0.2 ml/min. The eluents for the positive polarity mode were eluent A (0.1% FA in Water) and eluent B (Methanol). The eluents for the negative polarity mode were eluent A (5 mM ammonium acetate, pH 9.0) and eluent B (Methanol). The solvent gradient was set as follows: 2% B, 1.5 min; 2–100% B, 12.0 min; 100% B, 14.0 min; 100–2% B, 14.1 min; 2% B, 17 min. Q ExactiveTM HF-X mass spectrometer was operated in positive/negative polarity mode with spray voltage of 3.2 kV, capillary temperature of 320°C, sheath gas flow rate of 40 arb and aux gas flow rate of 10 arb.

### Processing and Statistical Analysis of Metabolomics Data

The raw data files generated by UHPLC-MS/MS were processed using the compound discoverer 3.1 (CD3.1, Thermo Fisher) to perform peak alignment, peak picking, and quantitation for each metabolite. The main parameters were set as follows: retention time tolerance, 0.2 min; actual mass tolerance, 5 ppm; signal intensity tolerance, 30%; signal/noise ratio, 3; and minimum intensity, 100,000. Peak intensities were normalized to the total spectral intensity. The normalized data was used to predict the molecular formula based on additive ions, molecular ion peaks and fragment ions. Then peaks were matched with the mzCloud (https://www.mzcloud.org/), mz Vaultand Mass Listdatabase to obtain the accurate qualitative and relative quantitative results. The identified metabolites were analyzed by multidimensional statistical and further to show the distribution of the original data and the classification of variables, including principal component analysis (PCA) was applied in all samples using R package models (http://www.r-project.org/) (Warnes et al., 2007), partial least squares-discriminant analysis (PLS-DA) was applied in comparison groups using R package ropls (http://www.r-project.org/) (Thevenot 2016), and orthogonal partial least squares discriminant analysis (OPLS-DA) was applied in comparison groups using R package models (http://www.r-project.org/) (Warnes et al., 2007). The characteristics of metabolite expression patterns were used to identify the differential abundance of the metabolites according to the criteria of a *p* value of the T test<0.05 and a variable importance in projection (VIP) value ≥ 1, which were indicated differential metabolites between different groups. The differential metabolites were annotated and subjected to enrichment analysis according to the KEGG metabolome database. An FDR≤0.05 defined pathways that were significantly enriched in differential metabolites. Pathway over-representation was evaluated by MSEA using the MetaboAnalyst module.

### Integrative Analysis of the Metabolome and Transcriptome

We examined and integrated the DEGs and DAMs for overlapping metabolic pathways performed by two way orthogonal PLS (O2PLS) analysis to identify hypoxia related genes and pathways based on both the metabolome and transcriptome datasets. In addition, metabolome and transcriptome data integration was calculated by using pearson correlation coefficients. The pairs of genes and metabolites in common metabolic pathways (with absolute Pearson correlation coefficient >0.995 and *p* < 0.05) were visualized using Cytoscape (V3.3.0). The top 50 gene and metabolite pairs were selected for heatmap analysis. Additionally, the top 250 pairs of genes and metabolites (with an absolute pearson correlation >0.5) were employed for metabolite transcript network analysis using R graph packages.

## Results

### Identification of DEGs in ATII Cells

**FIGURE 1:**
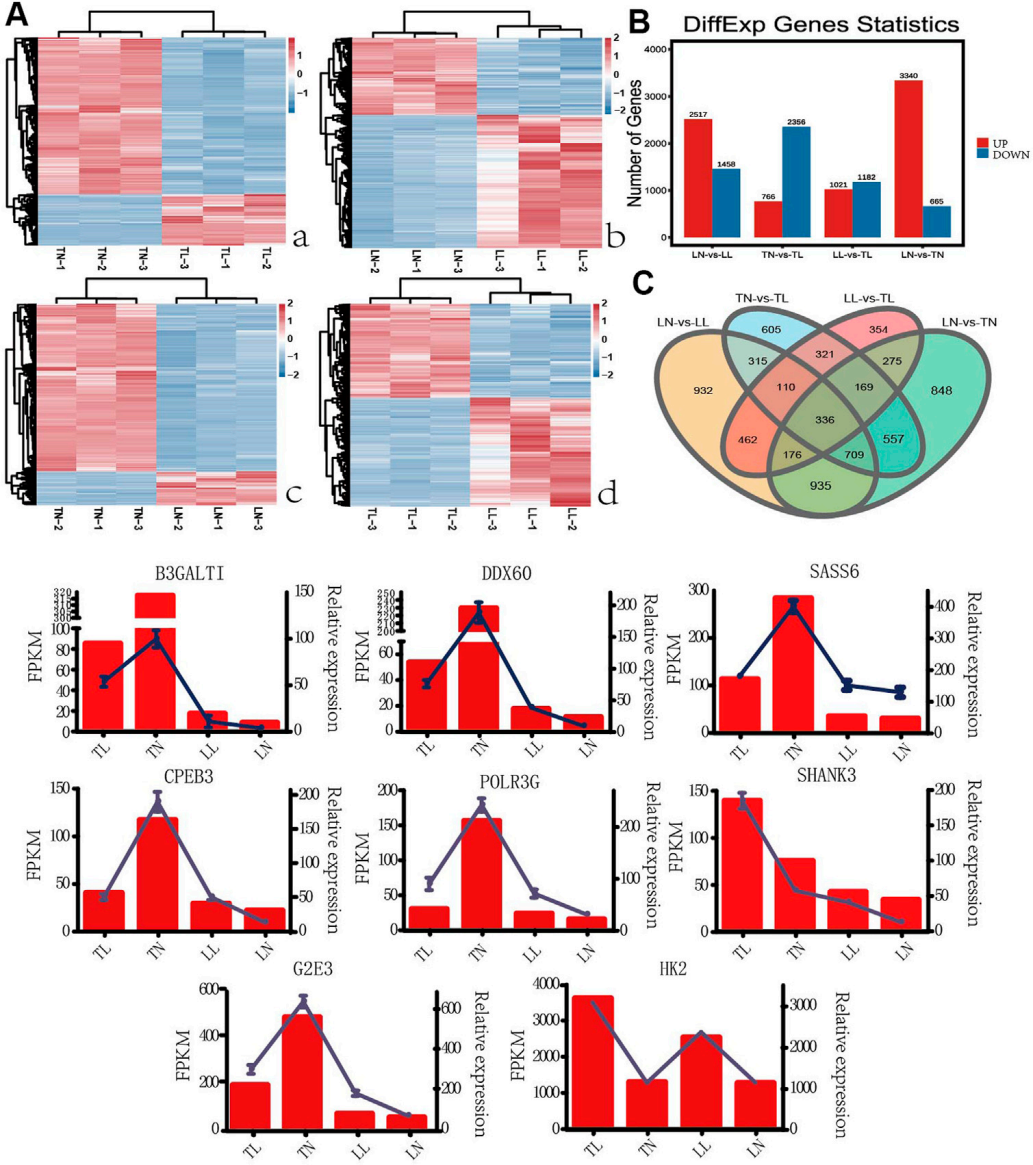
EDGs expression results among the four groups by RNA-seq. **(A)** The heatmap shows the relative expression patterns of DEGs among the four groups. Each column represents a sample, and each row represents the expression levels of a single mRNA in various samples. The color scale of the heat map ranges from blue (low expression) to red (high expression). **(a)** Heatmap of DEGs for TN and TL groups. **(b)** Heatmap of DEGs for LN and LL groups. **(c)** Heatmap of DEGs for LN and TN groups. **(d)** Heatmap of DEGs for LL and TL groups. **(B)** The histogram shows the number of DEGs identified among the four groups. **(C)** Venn diagram of DEGs interactions based on the overlapping DEGs among the four groups. **(D)** Expression patterns of eight randomly selected DEGs. Histogram represent the change in transcript level according to the FPKM value of RNA-seq (left y-axis), and Broken line indicate that relative expression level defense by RT-PCR (right y-axis).

**FIGURE 2:**
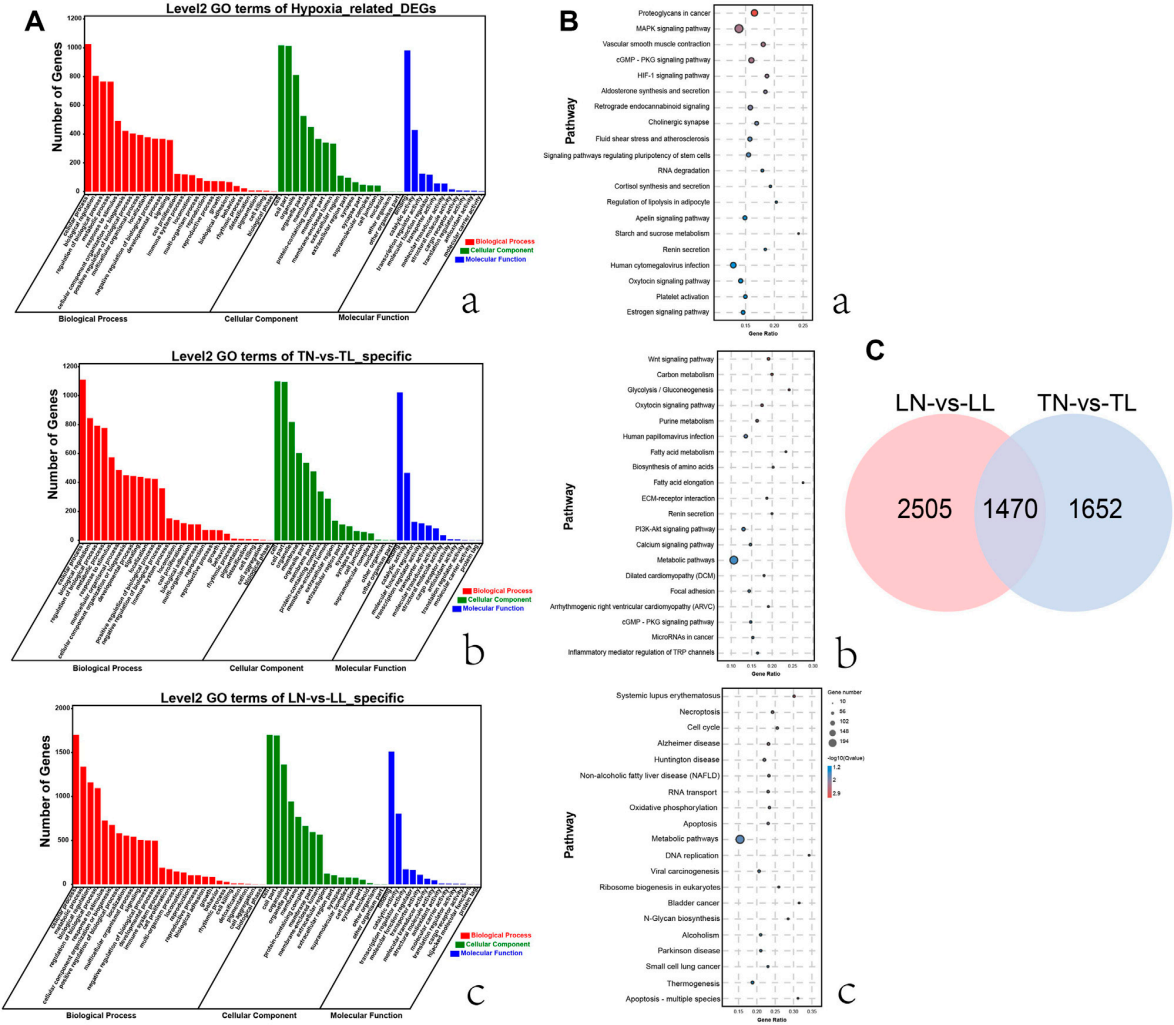
Functional annotation analysis of DEGs in ATII cells among the four groups. **(A)** Histogram of GO annotation results of DEGs. The abscissa is the second level GO term, and the ordinate is the number of DEGs in the term. **(a)** GO annotation results of 1,470 DEGs shared for normoxic (TN and LN) and hypoxic (TL and LL) groups. **(b)** GO annotation results of 1,652 specific DEGs (excluding DEGs shared between normoxia and hypoxia groups) for TN and TL groups. **(c)** GO annotation results of 2,505 specific DEGs (excluding DEGs shared between normoxia and hypoxia groups) for LN and LL groups. **(B)** Top 20 KEGG enrichment pathways of DEGs. The ordinate is the pathway, and the abscissa is the enrichment factor. Darker colors indicate smaller q-values. **(a)** Pathway enrichment analysis of DEGs for normoxic (TN and LN) and hypoxic (TL and LL) groups. **(b)** Pathway enrichment analysis of 1,652 specific DEGs (excluding DEGs shared between normoxia and hypoxia groups) for TN and TL groups. **(c)** Pathway enrichment analysis of specific DEGs (excluding DEGs shared between normoxia and hypoxia groups) for LN and LL groups. **(C)** Venn diagram of hypoxia related DEGs interactions based on the overlapping DEGs between normoxic (TN and LN) and hypoxic (TL and LL) groups.

To obtain an overview of the ATII cells transcriptome in normoxic and hypoxic environments, raw data (Q30 > 93.13%, with clean read ratio of >95.61%) were obtained for RNA-Seq (Supplementary Figure S1). Twelve libraries generated an average of 74,968,018 clean paired reads, and 95.61–98.28% of the clean reads were sequentially mapped to the porcine reference genome by Illumina paired-end sequencing technology (Table 1). Overall, 3,975 (2,517 up-regulated; 1,458 down-regulated), 3,122(766 up-regulated; 2,356 down-regulated), 2,203 (1,021 up-regulated; 1,182 down-regulated) and 4,005 (3,340 up-regulated; 665 down-regulated) DEGs were identified in the LN vs. LL, TN vs. TL, LL vs. TL, and LN vs. TN groups, respectively (Figures 1B,C, Supplementary Table S2 in Supplementary Material S3). A clustering heatmap analysis of these mRNAs showed excellent repeatability and gene expression profiles among the four groups (Figure 1A). A total of 1,470 DEGs were identified and screened based on the intersection in ATII cells between normoxic (TN, LN) and hypoxic (TL, LL) groups to explore the potential functions of gene responses in hypoxia, which considered as the most interesting candidates (Figure 2C, Supplementary Material S4). Furthermore, 54 novel genes were identified in the sequencing data. Eight mRNAs were randomly selected and identified using qRT-PCR to validate the accuracy of the sequencing data, which indicated that the qRT-PCR results were consistent with the mRNA-seq data (Figure 1D).

**TABLE 1:** Overview of the reads and quality filtering of the twelve libraries.

Sample	RawData (bp)	CleanData (bp)	AF_Q20 (%)	AF_Q30 (%)	AF_GC (%)	Total_Mapped (%)
LL-1	9360523800	9130040100	98.00%	94.14%	45.26%	33162515 (54.61%)
LL-2	3844967700	3676299300	98.25%	94.80%	45.14%	17384372 (71.20%)
LL-3	6975536100	6726007800	97.98%	94.35%	46.00%	28245513 (63.28%)
LN-1	9269896500	8997964200	97.85%	93.99%	47.17%	38467100 (64.60%)
LN-2	9584649000	9324353400	97.68%	93.63%	47.49%	40264448 (65.30%)
LN-3	10133538900	9857985900	97.83%	93.91%	46.96%	4,2831845 (65.65%)
TL-1	15305644800	14958605400	97.83%	93.95%	47.43%	69004867 (69.61%)
TL-2	14832318000	14515924800	97.90%	94.05%	47.02%	67328250 (70.03%)
TL-3	13664940600	13355162400	97.85%	93.82%	46.92%	60049093 (67.93%)
TN-1	17563924500	17262325500	97.65%	93.70%	47.33%	90569832 (79.03%)
TN-2	13469701500	13203415200	97.57%	93.45%	47.04%	70629189 (80.58%)
TN-3	14186481600	13934348700	97.84%	94.16%	47.14%	73733028 (79.71%)

### Enrichment and Functional Annotation of Differential Gene Expression in Response to Hypoxia

According to GO annotation, DEGs in four groups were classified into three major categories including biological process, cellular component and molecular function (Supplementary Figure S2). Comparing 1,470 DEGs of 2% O_2_ (TL, LL) groups with the 21% O_2_ (TN, LN) groups revealed that biological process was the most enriched category, with a total of 54 terms, followed by the cellular component and molecular function categories. In addition, cellular process, cell, and binding were the most abundant terms in biological process, cellular component and molecular function, respectively (Figure 2A). Interestingly, 1,025 DEGs between normal oxygen groups and hypoxia groups were mainly enriched in the cellular process term (GO: 0009987) of biological process. GO: 0005634, GO: 0005488, and GO: 0019219 were associated with the nucleus, binding, and regulation of nucleobase-containing compound metabolic processes were most significantly enriched between the normoxia and hypoxia groups. The top 20 pathways with the most significant enrichment among four groups were identified (Supplementary Figure S2). Multiple DEGs shared between the normoxia (TN, LN) and hypoxia groups (TL, LL) were enriched KEGG pathways categories, among which the most significant enriched category was proteoglycans, followed by MAPK signaling and then vascular smooth muscle contraction (Figure 2B). In addition, signal transduction of environmental information processing, global and overview maps of metabolism, endocrine system of organismal system, cellular community-eukaryotes of cellular process and folding, sorting and degradation of genetic information processing were also the most enriched pathways, respectively.

Interesting, system development, anatomical structure development, and multicellular organism development terms significant enriched by specific DEGs (excluding DEGs shared between normoxia and hypoxia groups) between the TN and TL groups, meanwhile, intracellular part, intracellular, and intracellular membrane-bounded organelle significantly enriched by specific DEGs (excluding DEGs shared between normoxia and hypoxia groups) between the LN and LL groups. Wnt signaling pathway, carbon metabolism and glycolysis/gluconeogenesis were most significantly enriched pathways by specific DEGs (excluding DEGs shared between normoxia and hypoxia groups) between the TN and TL groups, however, systemic lupus erthematosus, necroptosis, and cell cycle were the most significantly enriched pathways by specific DEGs (excluding DEGs shared between normoxia and hypoxia groups) between the LN and LL groups.

### Differentially Accumulated Metabolites Analysis

**FIGURE 3:**
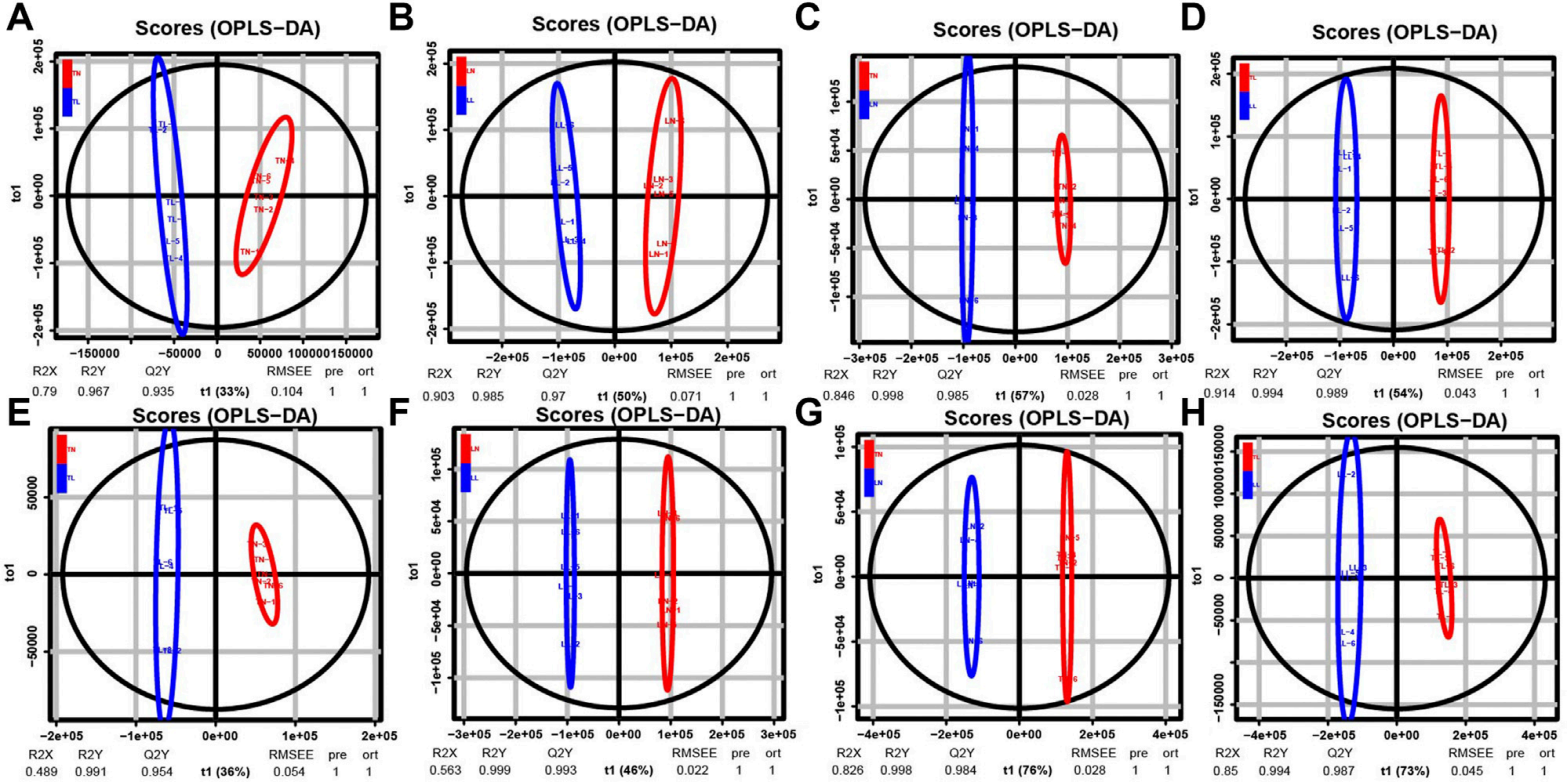
OPLS-DA scores of polar components of ATII cells metabolic profiling analysis. The OPLS-DA models were derived from the LC-MS metabolomic profiles among four groups. Both negative mode (NEG) are shown for **(A–D)**, and positive (POS) models are shown for **(E–H)** as indicated.

**FIGURE 4:**
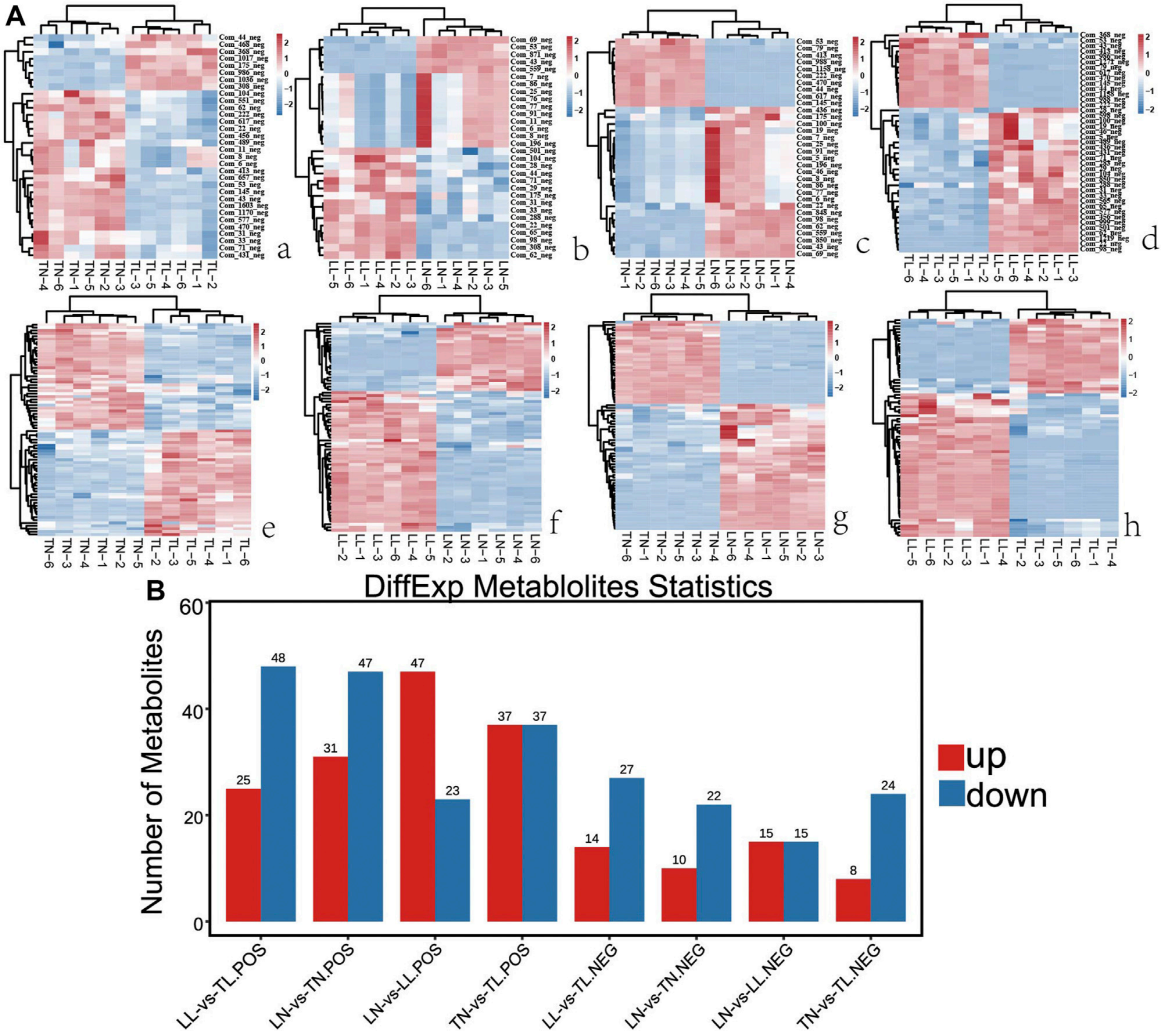
Comparative analysis of DAMs expression among the four groups by metabolomic. **(A)** The heatmap shows the relative expression patterns of DAMs among the four groups. Each column represents a sample, and each row represents the expression levels of a single metabolomic in various samples. The color scale of the heat map ranges from blue (low expression) to red (high expression). **(a)** Heatmap of DAMs for TN and TL groups. **(b)** Heatmap of DAMs for LN and LL groups. **(c)** Heatmap of DAMs for LN and TN groups. **(d)** Heatmap of DAMs for LL and TL groups. **(B)** The histogram shows the number of DAMs identified among the four groups.

Nontarget metabolomics was used to profile the potential impact of hypoxic environment on the metabolome of ATII cells. A PCA score plot showed the distributions of origin data, and the samples were separated accordingly (Supplementary Figure S3Aa, Bb). The PCA score plots of the TN-TL (PC1 = 36.8%, PC2 = 31.3%) group in positive (POS) mode were analyzed, and the score plots of PCA based on the TN-TL (PC1 = 50%, PC2 = 29.5%) group in negative (NEG) mode were revealed (Supplementary Figure S3 in Supplementary Material S5). Indeed, the PCA results illustrated that TN, TL, LN, and LL were clearly separated according to the corresponding groups. PLS-DA and OPLS-DA revealed that the TN, TL, LN, and LL groups were clearly distinguished from each other, which illustrated that the differences in metabolite accumulation among the four groups were significant (Figure 3). A total of 74 (37 up- and 37 down-regulated) and 32 (8 up- and 24 down-regulated) DAMs were identified between the TN and TL groups in POS and NEG modes, respectively (Figure 4; Supplementary Table S3, Supplementary Figure S4 in Supplementary Materials S6).

**FIGURE 5:**
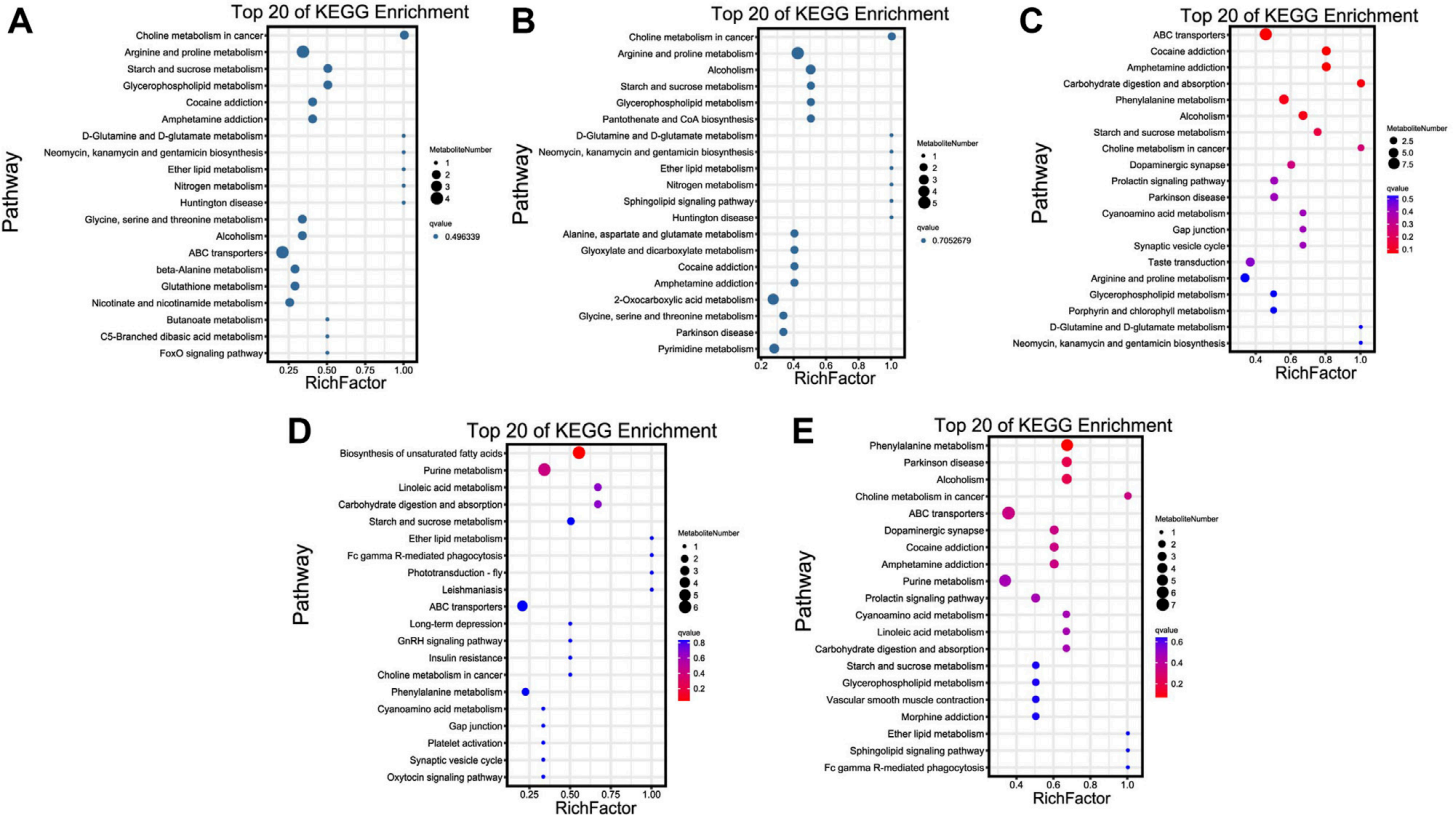
Top 20 KEGG enrichment pathways of DAMs. The ordinate is the pathway, and the abscissa is the enrichment factor. Darker colors indicate smaller q-values. **(A)** Pathway enrichment analysis of DAMs for normoxic (TN and LN) and hypoxic (TL and LL) groups. **(B)** Pathway enrichment analysis of DAMs for TN and TL groups. **(C)** Pathway enrichment analysis of DAMs for LN and LL groups. **(D)** Pathway enrichment analysis of DAMs for TN and LN groups. **(E)** Pathway enrichment analysis of DAMs for TL and LL groups.

KEGG enrichment results revealed that most of these metabolites were significantly enriched in choline metabolism in cancer, ABC transporters, biosynthesis of unsaturated fatty acids, and phenylalanine metabolism pathways of TN vs. TL, LN vs. LL, LN vs. TN, and LL vs. TL, respectively (Figure 5). Choline metabolism in cancer, and arginine and proline metabolism were most significant enriched pathways between normoxic (TN, LN) and hypoxic (TL, LL) groups.

### Integrated Analyses of Hypoxia-Related Pathways by Metabolome and Transcriptome

**FIGURE 6:**
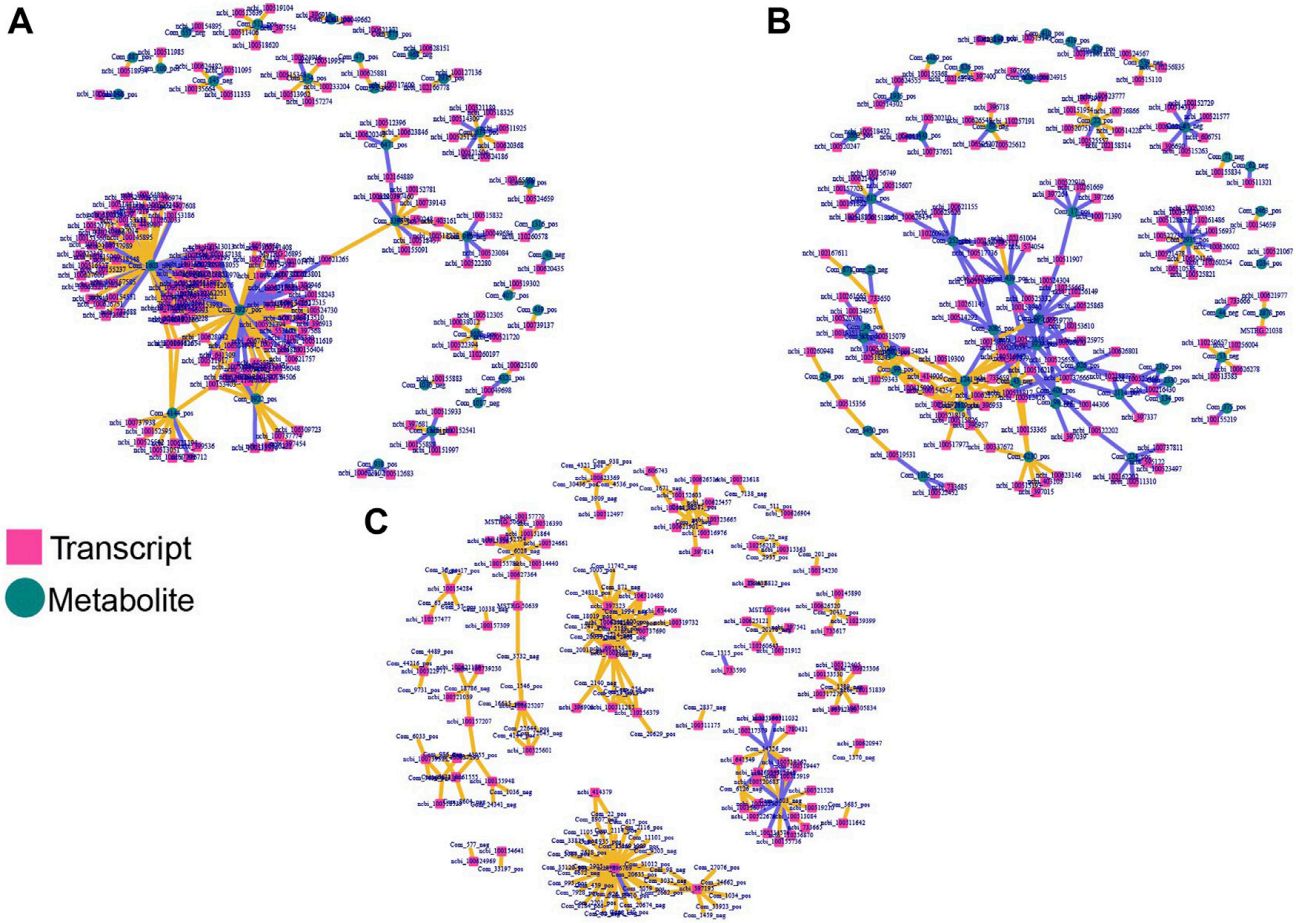
Integrated network analyses of DEGs and DAMs. The square nodes represent transcripts, and the circle nodes represent metabolites. **(A)** Co-expression network analyses of DEGs shared for normoxic (TN and LN) and hypoxic (TL and LL) groups and DAMs. **(B)** Co-expression network analyses of specific DEGs (excluding DEGs shared between normoxia and hypoxia groups) and DAMs for TN and TL groups. **(C)** Co-expression network analyses of specific DEGs (excluding DEGs shared between normoxia and hypoxia groups) and DAMs for TN and TL groups.

To obtain comprehensive insight and explore the correlation between hypoxia related DEGs and DAMs, the DEGs were analyzed in the form of venn diagrams (Figure 1). KEGG assessment indicated that DEGs between normoxic (TN, LN) and hypoxic (TL, LL) groups and DAMs were involved in the MAPK signaling pathway, vascular smooth muscle contraction and cGMP–PKG signaling pathway; specific DEGs (excluding DEGs shared between normoxia and hypoxia groups) and DAMs identified between TN and TL were mainly significantly enriched in huntington disease, metabolic pathways, and alcoholism pathways, while specific DEGs (excluding DEGs shared between normoxia and hypoxia groups) and DAMs between LN and LL were mainly significantly enriched in carbon metabolism and purine metabolism pathways (Supplementary Material S7). The gene–metabolite correlation pairs with absolute pearson correlation coefficient >0.995 and *p* < 0.05 were used to build hypoxia related regulatory networks. Through the integration of the hypoxia-related genes and metabolites identified in ATII cells during hypoxia, potentially regulatory for mRNA–metabolite pairs were analyzed based on the filtered genes. Three integrated analysis and coexpression networks were constructed for DEGs between normoxic (TN, LN) and hypoxic (TL, LL) groups and DAMs, specific DEGs (excluding DEGs shared between normoxia and hypoxia groups) and DAMs identified during the ATII cells response to hypoxia of Tibetan pigs (TN and TL) and Landrace pigs (LN and LL) under different oxygen levels, and the top 300 relationship pair network diagrams are presented (Figure 6). Guanosine-3′, 5′-cyclic monophosphate (com-1603-neg), Gly-Tyr (com-471-pos), and phenylacetylglycine (com-43-neg) were significantly correlated with a large number of different transcripts respectively. On the contrary, ncbi-396769 were significantly correlated with a large number of metabolites. Sulfamethazine (com-3927-pos), Guanosine-3′, 5′-cyclic monophosphate (Com-1603-neg), and kynurenic acid (com-3922-pos) were significantly correlated with a large number of specific DEGs (excluding DEGs shared between normoxia and hypoxia groups) between the TN and TL, meanwhile, 2,3,4,9-Tetrahydro-1H-β-carboline-3-carboxylic acid (com-2935-pos), YMH (com-1241-pos), and D-Raffinose (com-617-pos) were significantly correlated with a large number of specific DEGs (excluding DEGs shared between normoxia and hypoxia groups) between the LN and LL.

## Discussion

A large number of metabolome sequences, providing data on important parameters related to hypoxia, were generated some time ago, and their generation accelerated after research on adaptation to hypoxia in native high-altitude species, including Tibetan sheep, yaks, and Tibetan pigs (He et al., 2019; Ma et al., 2019; Du et al., 2021). The morphology of pulmonary artery cast specimens, the values of blood physiological indexes and the expression network of hypoxia-responsive genes were identified in Tibetan pigs compared to Landrace pigs in our previous studies (Yang et al., 2021a; Yang et al., 2021b). It can be assumed that changes in DEGs and DAMs reflect the hypoxic adaptation mechanism by regulating the expression of genes and metabolism to benefit ATII cell regeneration and transdifferentiation. In the present study, we focused on the hypoxia adaptation of ATII cells in pigs and identified 546 genes and metabolites with differential abundance between normal-altitude and high-altitude of Tibetan pigs and Landrace pigs. GO enrichment analysis revealed that hypoxia-related DEGs were associated with the nucleus, binding, and the regulation of nucleobase-containing compound metabolic processes. Moreover, several hypoxia-related signaling pathways, such as the MAPK signaling pathway, vascular smooth muscle contraction and the cGMP-PKG signaling pathway, which may form a complex cascade of responses to promote the regeneration and transdifferentiation of ATII cells, are active in hypoxic environments to protect pulmonary normal function.

### Speculation of the Hypoxia Signaling Pathway Affecting the Regulation of ATII Cells

As we all known, HIF-1α protein synthesis can result in the activation of MAPK pathways through interaction with their cognate receptor tyrosine kinases (Shin et al., 2010; Olson and Vliet, 2011). MAPK is a member of the family of enzymes involved in oxygen sensing and plays an important role in the mechanism underlying the increased tolerance of the bighead carp heart to acute hypoxia (Zhou et al., 2020). In this study, cAMP (Com_18786_neg) was found to be increased in the hypoxia groups and showed a strong correlation with hypoxia-related DEGs (above 300); cAMP may activate MAPK signaling to promote cell clone formation and proliferation in alveolar epithelial cells under hypoxia. Similar results have been found in periodontal ligament stem cells (He et al., 2016; Kabiri et al., 2018). Previous studies have shown that the sodium hydrosulfide-mediated inhibition of the cigarette smoke-induced phosphorylation of ERK, JNK and p38 MAPK may be a novel strategy for the treatment of chronic obstructive pulmonary fibrosis through the inhibition of inflammation, epithelial cell injury and apoptosis (Guan et al., 2020; Shologu et al., 2018; Singh et al., 2018). Therefore, we focused our further studies on MAPK signaling. Numerous hypoxia-related DEGs and metabolites of ATII cells identified between the TN and TL groups were enriched in the MAPK pathway; thus, the activation of MAPK pathways may arise under exposure to hypoxia and play critical roles in opposing the inflammatory response and regulating cell proliferation, differentiation, and apoptosis (Dong et al., 2019; Estaras et al., 2021). Through an in-depth analysis of the DEGs and the metabolite interaction network, we found that interactors were enriched in the MAPK pathway and could further regulate other pathways to promote the hypoxia-induced transdifferentiation of ATII cells (Xia et al., 2018; Wang et al., 2019). Taken together, these results suggested that MAPK signaling may contribute to suppressing the effect of hypoxia-induced inflammation and alveolar epithelial cell injury.

### Effects of Hypoxia on Transcription Expression and Metabolite Formation in ATII Cells

Multiple lines of evidence have demonstrated that cyclic GMP (cGMP) mediates the action of natriuretic peptides (NPs) and nitric oxide (NO) as intracellular second messengers that regulate a broad array of physiological processes (Tsai and Kass, 2009). The PKG1 isoform-specific activation of established substrates leads to a reduction in the cytosolic calcium concentration and/or a decrease in the sensitivity of myofilaments to Ca2+, resulting in smooth muscle relaxation (Adebola and Usha, 2011; Rainer and Kass, 2016). The cGMP-PKG signaling pathway could contribute to the attenuation of hyperlipidemia by Lei-gong-gen formula granules in rats, and this pathway has long been known to be a crucial therapeutic target in injury (Braun et al., 2018; Lan et al., 2020). As expected, we also discovered that a number of hypoxia-related DEGs and metabolic pathways were significantly enriched in cGMP–PKG signaling pathway by analyzing the results of KEGG pathway enrichment, for example, adenosine 5′-phosphate (com_27274_pos), adenosine (com_190_pos) and MADS-box transcription enhancer factor 2C (*MEF*2*C*), that may provide the cellular protection and serve as a potential therapeutic target for protect cell against injury of hypoxia (Mahneva et al., 2019). Likewise, PPARγ antagonizes the hypoxia-induced activation of hepatic stellate cells by cross-mediating the PI3K/AKT and cGMP/PKG signaling pathways (Zhang et al., 2018a). As a result, the higher expression of cGMP observed in ATII cells in the hypoxia groups (LL, TL) may exert physiological action mediated by two forms of cGMP-dependent protein kinase (PKGs), cGMP-regulated phosphodiesterases and cGMP-gated cation channels, among which the PKGs might be the primary mediator (Inserte and Garcia-Dorado, 2015; Olivares-González et al., 2016). Aldosterone is secreted from the adrenal cortex and plays a vital role in water, salt and sodium homeostasis, thereby contributing to blood pressure control, which requires tight regulation of its secretion (Bollag, 2014). Aldosterone production by zona glomerulosa cells is enhanced by hypoxia via the promotion of the hydrolysis and accumulation of cholesterol ester in lipid droplets (Yamashita et al., 2020). Consistent with previous studies, we observed that hypoxia related DEGs and DAMs were greatly enriched in aldosterone synthesis and secretion and that the expression of adenosine was significantly higher in the TL group than in the TN group, which may result in various regulatory mechanisms of cAMP and circulating aldosterone levels, especially plasma potassium levels and the renin–angiotensin system (Bollag, 2014).

### Differential Regulation of Glycolysis and *MCM* of Tibetan Pigs Response to Hypoxia

**FIGURE 7:**
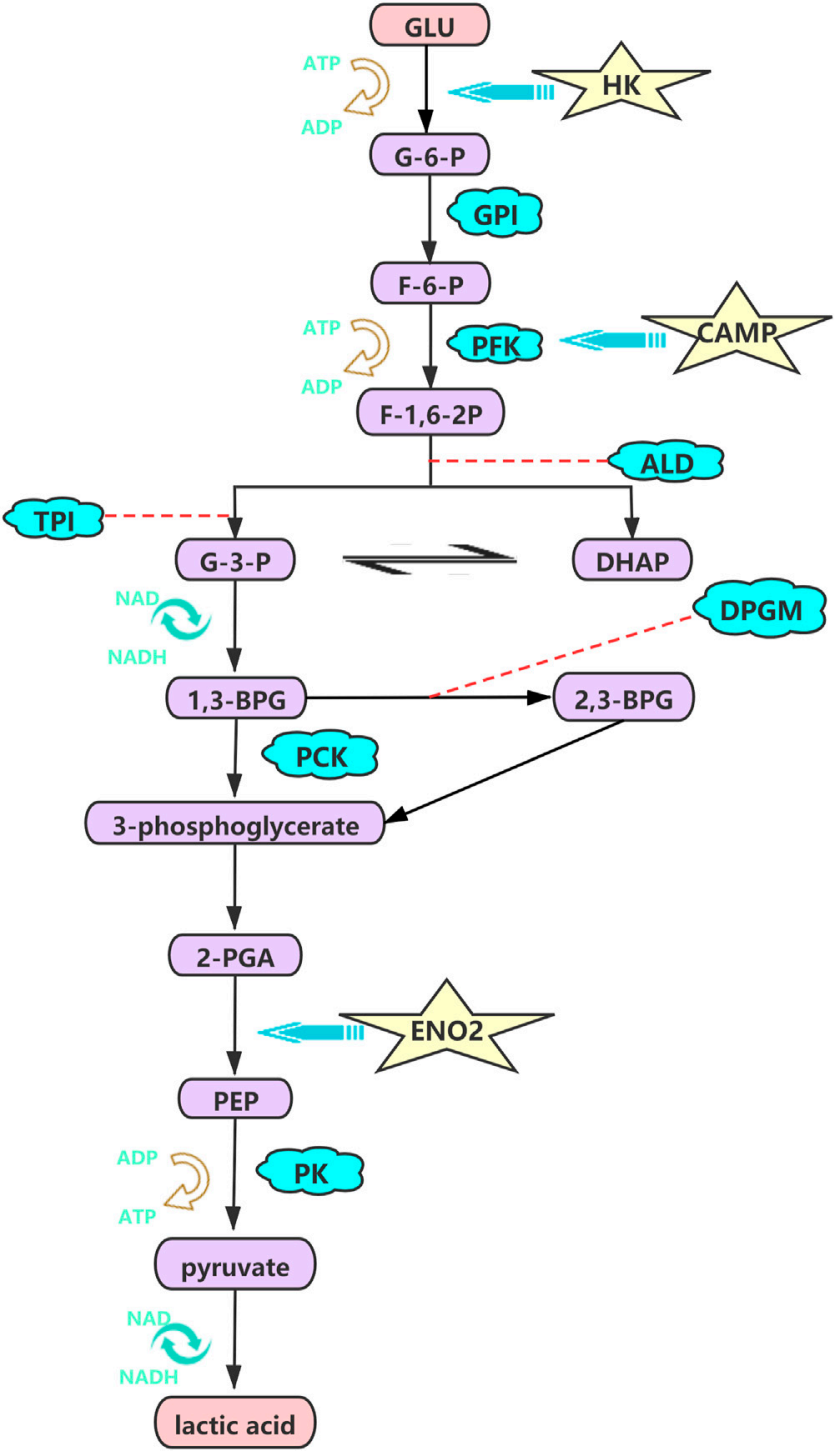
HK, CAMP, ENO2 were high regulated and promote the glycolysis, glucocorticoid resistance, and cell growth processing under hypoxia. The pathway was visualized by the adobe illustrator software.

We subsequently investigated the contribution of the components of HIF-1 signaling pathways, which can elevate interstitial pressure and protect cells from hypoxic injury through glucose metabolism, mitochondrial function and cell apoptosis, as potential therapeutic proangiogenic molecules (Balogh et al., 2019; Nagao et al., 2019; Li et al., 2021). The hypoxic metabolic switch could be regulated by hypoxia inducible factor-1α (*HIF*-1α), which induces glycolytic enzymes at the transcriptional level (Kierans and Taylor, 2021). Likewise, hexokinase (HK) mediates the first glycolytic enzymatic step and is key rate-limiting enzyme in glycolysis. The gene expression levels of *HK*1 and *HK*2 were significantly higher in the hypoxia groups (2% O_2_) than in the normoxia groups (21% O_2_) in our study, and the same pattern has been observed in pulmonary artery smooth muscle cells (PASMCs) (Chen et al., 2018a). The increase in glycolytic metabolism under hypoxia (TL and LL) may require the O_2_-independent glycolytic pathway to preferentially produce sufficient ATP to satisfy bioenergetic requirements, which profound effects on cellular physiology and hypoxic immunity in ATII cells under hypoxia (Semenza, 2017; Allen et al., 2020). Similarly, our results indicated that the expression of *ENO*2, the target gene of *HIF*, was significantly higher in the hypoxia (TL and LL) groups and promoted glycolysis, glucocorticoid resistance, and cell growth (Figure 7) (Liu et al., 2018).

Previous research showed that Tibetan pigs living in high-altitude environments have lower hemoglobin levels than those living in low-altitude environments, which indicates a blunted erythropoietic response to hypoxic challenge (Yang et al., 2021a). *HMOX*2, is involved in heme catabolism, showed significantly higher expression in TN than in TL (although the difference between LN and LL was not significant), which may lead to a more efficient breakdown of heme and help maintain a relatively low hemoglobin level in Tibetan pigs in a hypoxic environment; Tibetan researchers have shown similar results (Landry et al., 2016; Yang et al., 2016). MCM proteins play crucial roles in DNA helicase, replication and cell proliferation functions, which could negatively regulate HIF-1 activity, in addition to mutually regulating MCMs (Aparicio et al., 2012; Hubbi et al., 2013; Joshi et al., 2016). Several DEGs, such as *MCM*2, *MCM*3 and *MCM*4, were enriched in the cell cycle pathway and showed significantly higher levels in the LN group than in the LL group, in accord with the previously reported finding that hypoxia can downregulate *MCM*2-7 mRNA simultaneously in HCT116 cell lines and endothelial cells to decrease proliferation as a fundamental physiological response to hypoxia (Hubbi et al., 2011; Kondo et al., 2017). However, the different between the TN and TL groups was not significant, which may explain why Tibetan pigs adapt to hypoxic environments better than Landrace pigs.

## Conclusion

In conclusion, we identified several molecular pathways showing adaptive changes in ATII cells through integrated metabolomic and transcriptomic analyses, revealing that MAPK signaling may affect hypoxia-induced inflammation and alveolar epithelial cell injury and that glycolytic metabolism in hypoxic environments may have profound effects on cellular physiology in ATII cells. These findings provide a novel perspective and direction for the future exploration of the regulatory mechanisms of pulmonary development and function in Tibetan pigs.

## Data Availability

The original contributions presented in the study are publicly available. This data can be found here: National Center for Biotechnology Information (NCBI) BioProject database under accession number PRJNA778032.

## Abbreviations

DEGs, differentially expressed mRNAs; DAMs, differentially expressed metabolomics; TN, ATII cells of Tibetan pigs were cultured under 21% O_2_; TL, ATII cells of Tibetan pigs were cultured under 2% O_2_; LN, ATII cells of Landrace pigs were cultured under 21% O_2._; LL, ATII cells of Landrace pigs were cultured under 2% O_2_
