# Supplementary material for: Transcriptome and Metabolome Integration Provides New Insights Into the Regulatory Networks of Tibetan Pig Alveolar Type II Epithelial Cells in Response to Hypoxia
Source: Front Genet. 2022 Jan 21;13:812411. doi: 10.3389/fgene.2022.812411 (PMC8814526; doi:10.3389/fgene.2022.812411)
Supplement: Supplementary file 3 [file DataSheet1.doc]

**Table S1 Primers used to detect DEGs in ATII cells of pigs by qRT-PCR**

| **Genes** | **Primer sequences (5'-3')** | **Annealing temperature/℃** |
| --- | --- | --- |
| *B3GALT1* | F: ATGTCAAGCAAGAAACATCTCC | 60 |
|  | R: TTTACAAAACCCCTTCACCA |  |
| *CPEB3* | F: CAACGACTTGAAAACAAATGAC | 60 |
|  | R: GACTGGGAGGTTTGGGATT |  |
| *DDX60* | F: AGAAGAACCCATCACCAATCA | 60 |
|  | R: ACTCGTTAGGAAAGGCAAATC |  |
| *G2E3* | F: GTCAAGTGGAATTTGGCAGAG | 60 |
|  | R: TCGTTTACATCGGGGTGC |  |
| *HK2* | F: GATGGGACAGAACACGGAG | 60 |
|  | R: CATGAAGTTAGCCAGGCACT |  |
| *POLR3G* | F: CAATGGCTGGGAATAAAGG | 60 |
|  | R: CAGGAAATAGTGGAGGTGGTT |  |
| *SASS6* | F: AATGTGGTTGAGGGTAGACTGA | 60 |
|  | R: TTTTGGCAGCACAAGGGT |  |
| *SHANK3* | F: GCCCGTGTCCTGCTTTT | 60 |
|  | R: TCCGAATCTTTGTGGGTCTT |  |
| *β-actin* | F: CAGTCGGTTGGATGGAGCAT  R: AGGCAGGGACTTCCTGTAAC | 60 |

**Table S2 Numbers of DEGs were identified.**

| **Comparison group** | **DEGs** | |
| --- | --- | --- |
|  | **Upregulated** | **Downregulated** |
| LN-vs-LL | 2517 | 1458 |
| TN-vs-TL | 766 | 2356 |
| LL-vs-TL | 1021 | 1182 |
| LN-vs-TN | 3340 | 665 |

**Table S3 Numbers of DAMs were identified.**

| **Comparison group** | **DAMs** | | | |
| --- | --- | --- | --- | --- |
|  | **POS** | | **NEG** | |
|  | **Up-regulated** | **Down-regulated** | **Up-regulated** | **Down-regulated** |
| LN-vs-TL | 25 | 48 | 14 | 27 |
| LN-vs-TN | 31 | 47 | 10 | 22 |
| LN-vs-LL | 47 | 23 | 15 | 15 |
| TN-vs-TL | 37 | 37 | 8 | 24 |
