# Supplementary material for: Transcriptome and Metabolome Integration Provides New Insights Into the Regulatory Networks of Tibetan Pig Alveolar Type II Epithelial Cells in Response to Hypoxia
Source: Front Genet. 2022 Jan 21;13:812411. doi: 10.3389/fgene.2022.812411 (PMC8814526; doi:10.3389/fgene.2022.812411)
Supplement: Supplementary file 6 [file DataSheet2.docx]

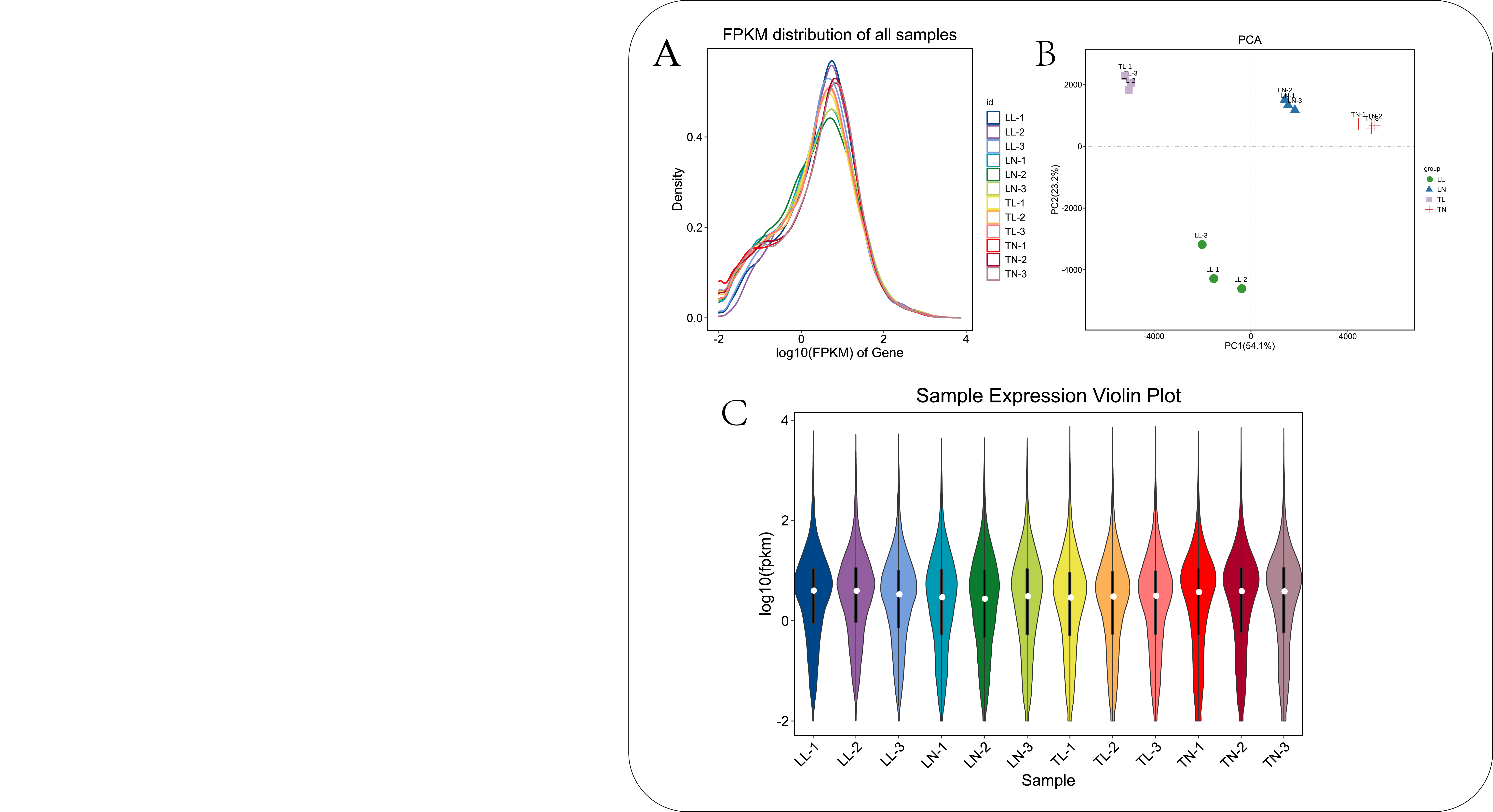


**Figure S1** A. Density plot of mRNA interactions based on the overlapping mRNAs among the four groups. B. A PCA score plot showed the distributions of mRNAs. C. DEGs among four groups. P-values and log2FC values were used to screen for differentially expressed transcripts according to the following thresholds: P < 0.05 and |log2FC| > 1.


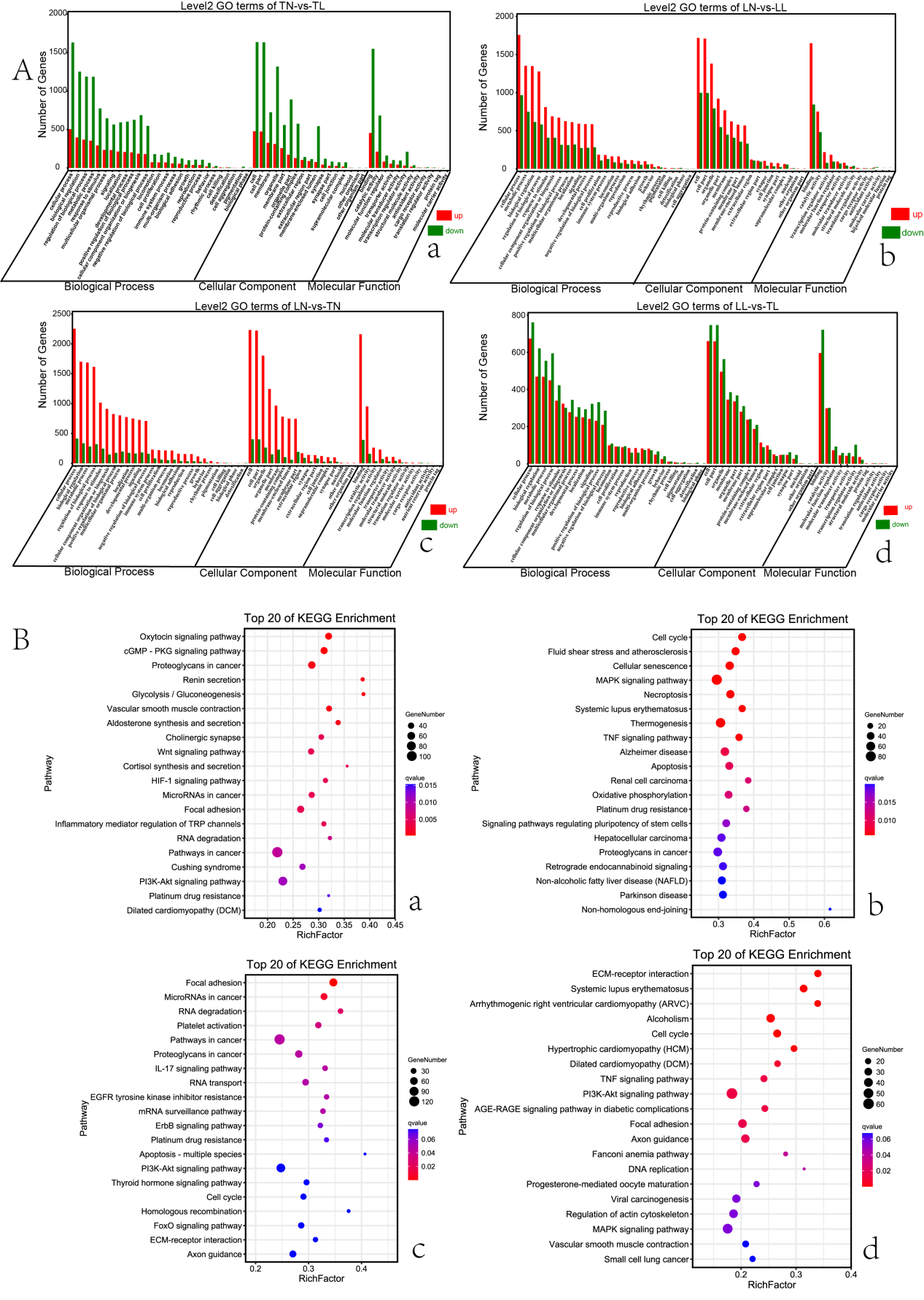


**Figure S2** Functional annotation analysis of DEGs in ATII cells among the four groups. **A.** Histogram of GO annotation results of DEGs. The abscissa is the second level GO term, and the ordinate is the number of DEGs in the term. **B.** Top 20 KEGG enrichment pathways of DEGs. The ordinate is the pathway, and the abscissa is the enrichment factor. Darker colors indicate smaller q-values. **a.** Pathway enrichment analysis of DEGs for TN and LN groups. **b.** Pathway enrichment analysis of DEGs for LN and LL groups. **a.** Pathway enrichment analysis of DEGs for TN and LN groups. **a.** Pathway enrichment analysis of DEGs for TL and LL groups.


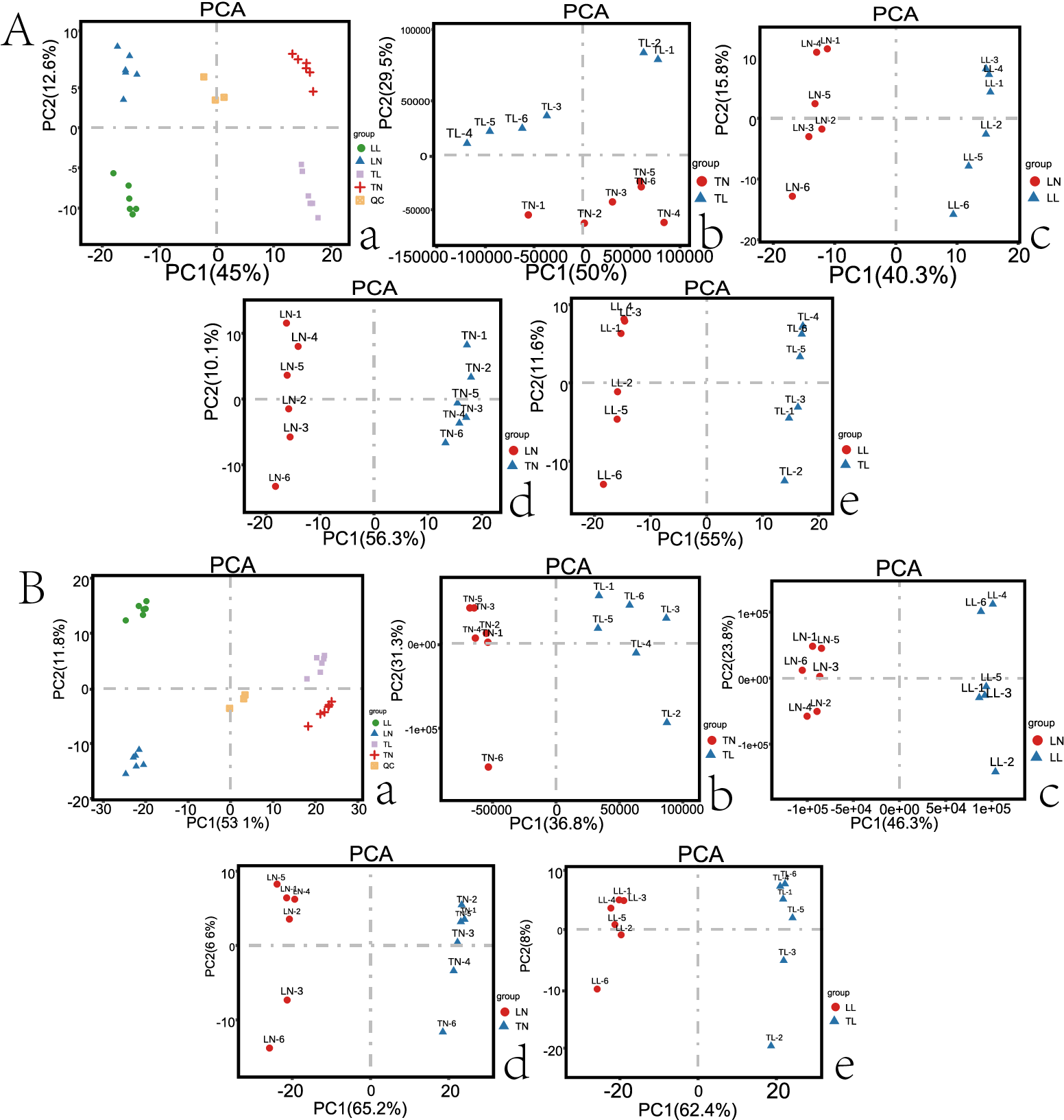


**Figure S3** PCA score plot of ATII cells metabolic profiling analysis. A. PCA score plot of negative mode (NEG) are shown for A. B. PCA score plot of positive mode (POS) are shown for B.


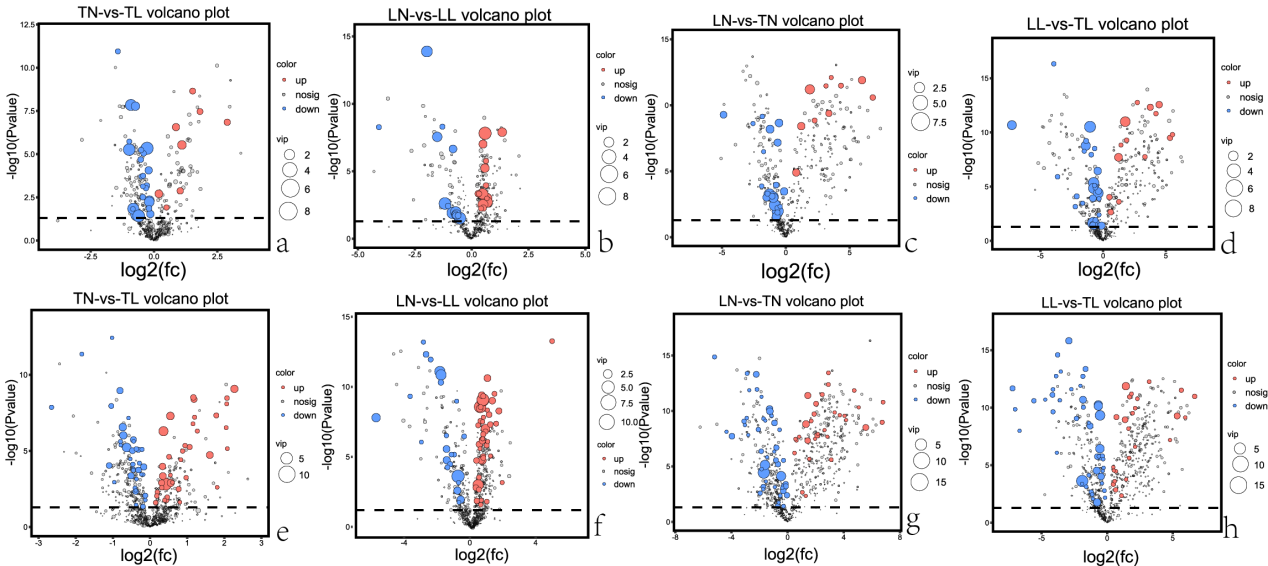


**Figure S4** Volcano plots of metabolites in the ATII cells among four groups. a-d. Volcano plots of metabolites were derived from the negative (NEG) models. e-h. Volcano plots of metabolites were derived from the positive (POS) models.
